# Pre-hospital telestroke and expanded hyper-acute telestroke network solutions to reduce geographic inequities: a brief review from the South Pacific

**DOI:** 10.3389/fstro.2024.1338003

**Published:** 2024-02-22

**Authors:** Anna Ranta, Heinrich J. Audebert, Luatupu Ioane-Cleverley

**Affiliations:** ^1^Department of Medicine, University of Otago, Wellington, New Zealand; ^2^Department of Neurology, Te Whatu Ora Health New Zealand Capital, Coast and Hutt Valley, Wellington, New Zealand; ^3^Department of Neurology and Center for Stroke Research, Charité Universitätsmedizin, Berlin, Germany; ^4^Department of Medicine, Te Whatu Ora Health New Zealand Capital, Coast and Hutt Valley, Wellington, New Zealand

**Keywords:** stroke, telestroke, telehealth, pre-hospital, indigenous, disparities, international, Pacific

## Abstract

Hyper-acute stroke treatments are time sensitive, and decision-making is complex. Telemedicine has been highly effective in breaking down regional access barriers by providing front line rural hospital clinicians with remote telemedicine decision support by remote stroke experts. With the advent of mechanical thrombectomy, hyper-acute stroke care has grown even more complex from both a decision-making and logistical perspective. Mobile Stroke Units (MSU) have been deployed in a few urban settings globally but are unlikely to address all global access issues due to geographical and logistical factors. This paper reviews the feasibility and benefit of extending telestroke into the pre-hospital setting as an adjunct or alternative to MSUs. It will discuss how this service model can fit into existing stroke networks and potential deployment strategies. Finally, the paper also considers potential scalability of pre- and in-hospital telestroke support across regional and international boundaries to further reduce global hyper-acute access inequities.

## Introduction

Stroke is a leading cause of death and disability in Aotearoa New Zealand (New Zealand) and globally (Collaborators GBDLRoS et al., 2018). Reperfusion therapies offer the greatest chance of stroke symptom reversal and disability free survival. However, treatment decisions are complex and highly time sensitive making them especially susceptible to inequitable access based on geographic barriers. Smaller, remote, and often socioeconomically disadvantaged populations have poorer on-site access to clinicians with stroke expertise, reperfusion decision-making, and provision of stroke interventions, which has been linked to poorer post-stroke outcomes (Thompson et al., 2020, 2022a). Additional barriers exist for some ethnic minorities (Thompson et al., 2022b).

We completed this review from a multi-ethnic South Pacific perspective. New Zealand consists of 16.5% indigenous Māori, 70% Europeans, 15% Asian, and 8% Pacific Island immigrants. Being a self-contained island nation with a publicly funded health system offers a relatively controlled environment to study new care systems while providing diverse ethnic and cultural insights, widening applicability. New Zealand, the size of the UK with a 10th of the population, ranks 21st for GDP among the 38 OECD countries (OECD, 2024), achieves very good health outcomes, and supports development in South Pacific Island neighbor nations (OECD Data, 2023).

## Hyper-acute stroke and telestroke care in New Zealand

**Figure 1:**
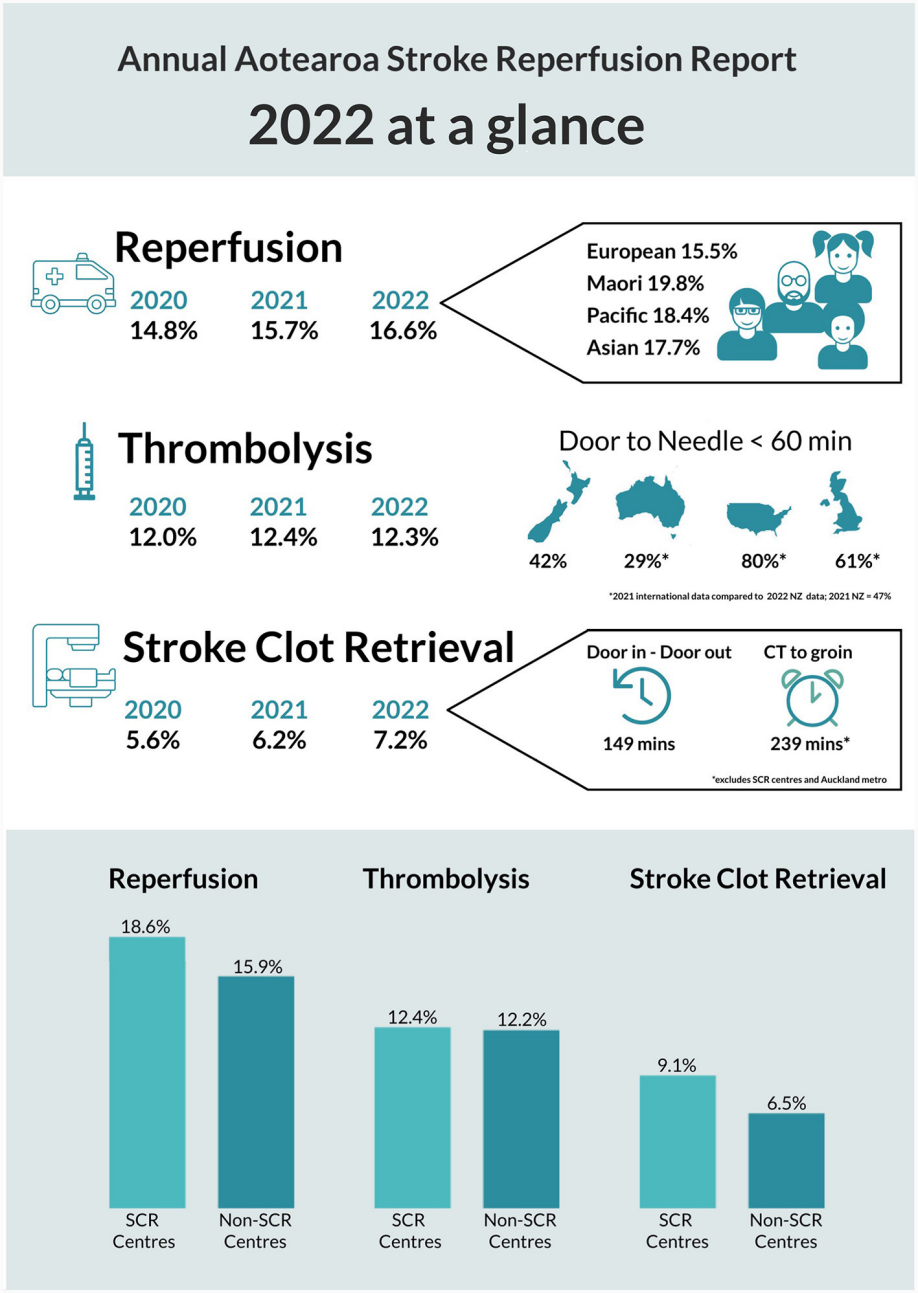
New Zealand acute stroke metrics 2022 national report summary. This is an official summary of a comprehensive annual report prepared for New Zealand Hospitals by the National Stroke Network operated by the New Zealand Ministry of Health. For clarity, SCR = Stroke Clot Retrieval (the official consumer endorsed term for thrombectomy in New Zealand); denominators include all hospitalized patients with ishaemic (ICD-10-AM I63) and stroke unspecified (ICD-10-AM I64) stroke during the report period regardless of other eligibility criteria or hospital arrival times. The ethnicity information and bar graphs at the bottom depict intervention rates by sub-group (e.g., ethnic sub-group or center type) and thus do not add to 100%. SCR or thrombectomy capable centers are excluded from the “Door in – Door out” and “CT-to-groin” times because these metrics specifically look at regional transfer delays.

In New Zealand, essentially all acute stroke patients are cared for at one of 30 secondary Computed Tomography (CT) capable public hospitals. Three of these hospitals offer thrombectomy services (Figure 1). CT capable hospitals may serve communities as small as 22,000 people; and some communities live >3 h drive from a CT capable hospital. The implementation of hospital-based hyper-acute telestroke services using videoconferencing to connect remote experts to rural hospital emergency department for expert decision support, has resulted in reductions of health inequities in New Zealand (Ranta et al., 2017; Hedlund et al., 2020). However, ongoing disparities exist. Firstly, many populations still live too far from Telestroke supported hospitals to access timely treatment. Secondly, the advent of mechanical thrombectomy has seen a new widening of the access gap because telestroke alone cannot provide this treatment to rural populations at their local hospital. And finally, variation in telestroke provision result in ongoing geographic access disparities for thrombolysis in some parts of New Zealand.

## Pre-hospital telestroke to improve equity

The distance from home to hospital creates what has seemed like insurmountable delays in reperfusion therapies. This held true until the advent of mobile stroke units (MSUs). MSUs are CT equipped ambulances that can literally take the treatment to the patients' door-step and this intervention has been shown to be highly efficacious and cost-effective (Fassbender et al., 2021; Kim et al., 2021; Chen et al., 2022; Turc et al., 2022). However, cost-efficacy is directly linked to the number of patients treated in an MSU requiring 100–150 thrombolysis eligible people to be assessed per year, is thus logistically limited to densely populated urban areas and is unlikely to address the issues faced by people living in remote rural areas (Fassbender et al., 2021).

As an alternative to the full MSU model, a “mini-MSU” model has been proposed where regular ambulances are equipped with telemedicine to allow a remote stroke expert to provide early diagnosis, triage, and guide medical interventions en route to hospital, making use of the in-ambulance time to reduce delays post hospital arrival. Pre-hospital telestroke has been shown to be feasible in pilot studies dating back to 2000 and recent improvements in cellular coverage and signal strength have greatly improved applicability (LaMonte et al., 2000; Bergrath et al., 2012; Belt et al., 2016; Barrett, 2017; Johansson et al., 2019). Reductions in treatment delays have been demonstrated (Espinoza et al., 2015; Kasab et al., 2021). One study reported a 20 min reduction in onset to imaging time (Espinoza et al., 2015; Brouns et al., 2016), another found a 17 min reduction in door-to-needle, and 87 min reduction in onset-to-groin times (Kasab et al., 2021).

Telemedicine's diagnostic accuracy has been demonstrated inside MSUs. As a result, telemedicine has replaced in-ambulance vascular neurologists in several locations reducing MSU operating costs and logistical limitations, although still precluding far reaching rural use (Geisler et al., 2019; Lumley et al., 2020).

More recently, a New Zealand based pre-hospital telestroke randomized controlled trial assessed diagnostic utility outside of the MSU setting (Scott et al., 2022). The trial found that pre-hospital telestroke was 100% (95% CI 90%−100%) accurate in predicting reperfusion candidates compared to pre-imaging emergency department-based diagnosis. By comparison, a Los Angeles Motor Score based large vessel occlusion (LVO) score achieved a 70.7% (95% CI 54.5%−83.9%) accuracy (*p* < 0.001). In predicting eventual thrombectomy intervention, telestroke was 88.6% (73.3-96.8) accurate and the score 56.1% (38.8–71.5; *p* = 0.005). It concluded that the utility of pre-hospital telestroke goes beyond a reduction in treatment delays by offering highly effective pre-hospital triage and bypass decision support.

This matters, because rural patients are particularly disadvantaged when transported to the nearest rural hospital—often without CT. By the time a patient has reached a CT capable hospital and then a thrombectomy center, many hours have passed even with the most efficient inter-hospital transport protocols. Bypassing smaller hospitals has been promoted making use of LVO paramedic scores. The RACECAT trial demonstrated reduced treatment delays for thrombectomy, but found no clear patient benefit (de la Ossa et al., 2022). The reason for this may be two-fold. Firstly, transfer times were already near optimal reducing the potential for benefit from direct transport. This is supported by a *post-hoc* analysis that found better outcomes at night when baseline delays were generally longer (García-Tornel et al., 2023). However, this association was only seen in imaging confirmed LVO patients underscoring that scores like RACE have limited accuracy in predicting LVO and eventual thrombectomy meaning some diversions will be inappropriate, a second potential explanation for the overall negative RACECAT trial (Patrick et al., 2021).

Based on the New Zealand trial, pre-hospital telestroke can reduce false positive thrombectomy candidates compared to generally sensitive, but less specific LVO scores. This may be because neurologists can also consider neurologic localization, stroke mimics, pre-morbid independence, medical history, timing, and patient consent. This allows better prediction of eventual thrombectomy beyond stroke severity/LVO detection. A neurologist will also be better able to assess whether forgoing closer thrombolysis over more distant thrombectomy is the best decision for a given patient based one individual patient, hospital, and geographic factors. Once a patient has been accurately diagnosed as a possible thrombectomy candidate and rapid access to thrombolysis is not considered more important, the patient can be transported to the nearest thrombectomy center bypassing all in-between rural and secondary hospitals. Ensuring patients who do not meet these criteria are transferred to nearer thrombolysis hospitals is important to avoid unproductive delays. In addition, non-strokes may not need even CT capable hospital care; the ‘highest' level of care is not always best—not only from a transport cost perspective but also considering the impact of displacement on patients and families.

In addition to improved specificity, it is very likely that telestroke neurologists also increase diagnostic sensitivity by more expertly localizing posterior circulation and distal anterior LVO clinical syndromes (e.g., isolated aphasia), however, this still requires confirmation.

**Figure 2:**
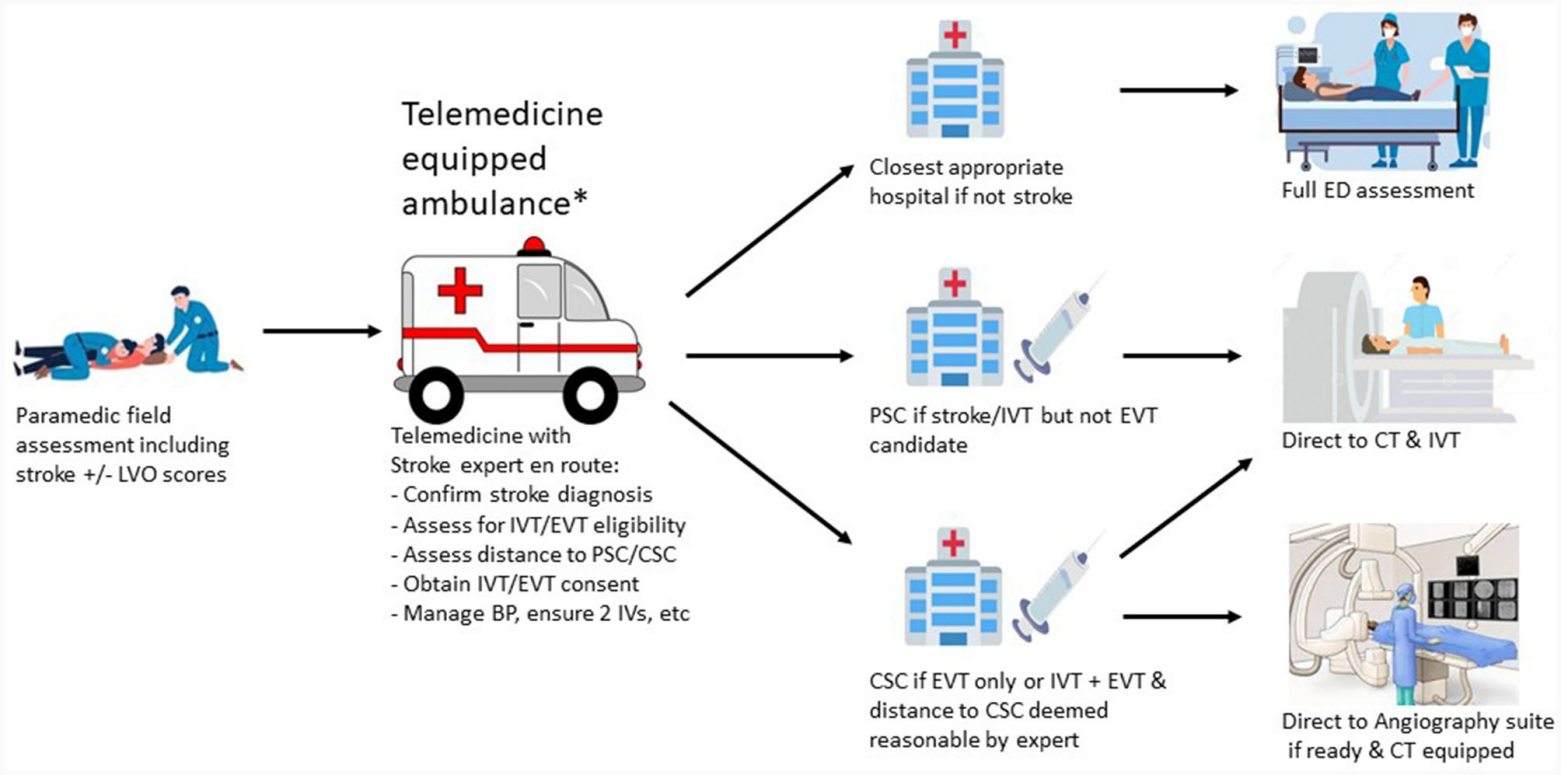
Telemedicine equipped ambulance workflow. *Transport may occur via helicopter if distance requires this especially in rural areas. LVO, large vessel occlusion; IVT, intravenous thrombolysis; EVT, endovascular thrombectomy; PSC, primary stroke center; CSC, comprehensive stroke center; BP, blood pressure; IV, itravenous catheter; ED, emergency department; CT, computed tomography.

Figure 2 shows the telemedicine ambulance equipped workflow.

## Implementing pre-hospital telestroke

New challenges arise when looking to deploy pre-hospital telestroke throughout a stroke network. These include governance, financial, staffing, and regional boundary challenges. A pre-hospital patient is under the care of the pre-hospital provider, but if a hospital-based neurologist offers advice it can become unclear as to who assumes overarching responsibility. Similarly unclear is who should remunerate pre-hospital neurologist involvement. This is especially relevant for out-of-region patients who may never present to the neurologist's own hospital. Inter-regional boundary crossing, bypassing secondary hospitals operating in separate networks, may also result in communication difficulties where networks use different telestroke providers, workflow tools, communication platforms, clinical guidelines, and referral criteria.

A logical solution to most of these issues is the implementation of a single super-regional or even national stroke and telestroke network aligning all clinical pathways, tools, rosters, and governance structures. Financial issues would also be easier to address once a single shared hospital centered network negotiates with pre-ambulance providers. Finally, inter-regional service variation and associated inequities can be addressed. In New Zealand a National Stroke Clot Retrieval Programme has brought together people from all relevant sectors including pre-hospital, emergency, inter-hospital, ICU, stroke, interventional, anesthesia, internal medicine, rural and urban hospital clinicians, managers and policymakers (Te Whatu Ora Health NZ, 2023). Nationally consistent thrombectomy referral, imaging, and inter-hospital transport guidelines have been developed. Telestroke service standards, key performance metrics, and neuro-interventionist training and standards have been agreed. A national neuro-interventionist fellowship has been implemented and single national imaging software and workflow procurement process is in the planning stages. Next steps entail implementation of a single national pre-hospital pathway incorporating both paramedic scores and pre-hospital telestroke. A single national telestroke and interventional service is currently being considered for funding (Te Whatu Ora Health NZ, 2023).

## Telestroke in the broader South Pacific

Beyond New Zealand and Australia, the South Pacific comprises numerous less-economically developed and diverse island states. Fiji has implemented an acute stroke team and is gearing up to start thrombolysis. Samoa is implementing organized stroke services with thrombolysis planned in 1–2 years and telemedicine support has been explored (Ioane-Cleverley, 2014). Both island nations are small with few specialists, but high stroke incidence. They have the requisite radiological infrastructure and the will to achieve optimal patient outcomes. Several solutions are being explored. Simply upskilling sufficient generalists in each country may not result in desired intervention rates (Ranta et al., 2017). Temporary support from New Zealand may not result in lasting benefits (Ranta and Busch, 2018). Ongoing remote support from New Zealand may create unsustainable and disempowering dependencies. Development of a spoke-to-spoke trans-Pacific telestroke network with 1–2 stroke specialists in each country linking up to provide reciprocal care may prove the most effective solution, but is complex (Ranta et al., 2016). Regardless of the chosen approach, service improvements should be driven by Pacific clinicians themselves, embracing Pacific strengths-based service delivery reflecting their culture, values and context. Efforts without understanding the local contexts and perspectives before ‘helping' can result in imposing a culturally incongruent model of care, despite best intentions.

## International telestroke

Several of the above solutions involve telestroke support crossing international boundaries, which is legally complicated, but achievable. International telestroke was feasible between New Zealand and Scotland (Ranta et al., 2016) and is operational between New Zealand and Australia. The link to Scotland offered the additional 12-h time zone difference benefit, meaning clinicians could manage remote night-time presenters during clinician day-time hours. The advantage of a single continent/region model is easier team development although these issues may become less and less important with increased globalization and widespread videoconferencing. It now seems increasingly conceivable that an international telestroke network could offer global coverage to the most remote and underserved areas and potentially also to developed countries to provide relief from night-time call outs. Our New Zealand group has considered physically stationing New Zealand neurologists in Europe for this purpose.

**Figure 3:**
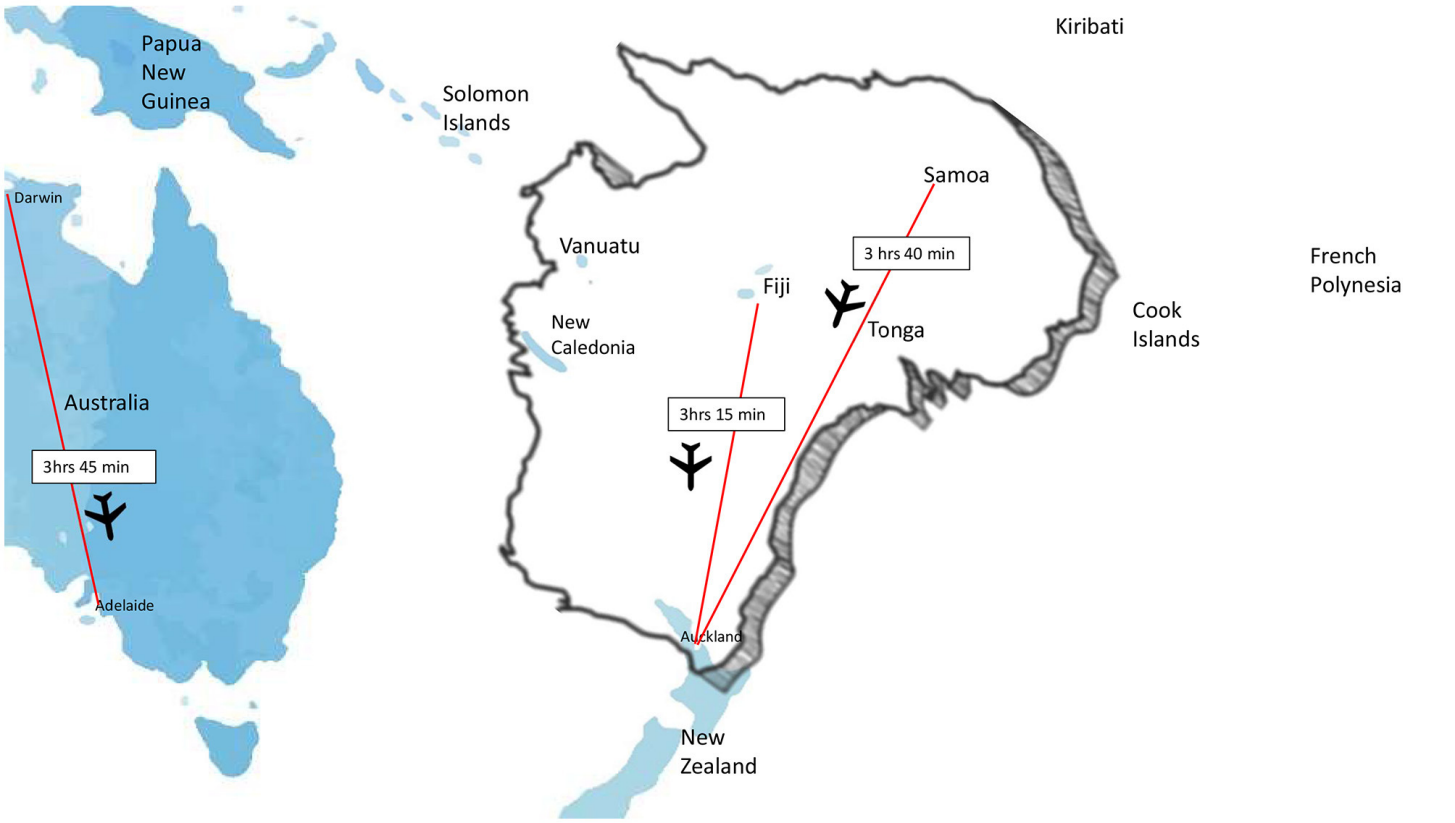
Between nation flight times in the South Pacific compared to longest flight time to a thrombectomy center within Australia.

The final barrier will be provision of thrombectomy in remote and under resourced countries. Training of local specialists is inconceivable in many locations and may be challenging to maintain if case volumes are small although should be explored wherever feasible. Alternatively, ultra-long transfers are possible and can still result in favorable outcomes as shown in an Australia-New Zealand case series (Garcia-Esperon et al., 2023). While this study described within country transfers, some of the reported distances were similar to distances between Pacific Islands and the nearest thrombectomy center in a neighboring country making this primarily a policy maker issue around cost recovery and medico-legal regulations. Such a model of care would ideally be supported by telestroke to ensure upfront optimal patient selection (Figure 3).

## Conclusion

Telestroke technology combined with collaborative clinical networking can result in substantial impact to reduce stroke care inequities. It is important to be aware of potentially increasing inequities as cutting-edge stroke therapies are implemented faster in some areas than others. As stroke care advances, it is imperative that innovation in implementation is similarly encouraged to ensure optimal access equity for all people with stroke globally. Finally, cultural awareness, competence, and facilitating self-determination are essential when supporting developing nations in this effort.

## Author contributions

AR: Conceptualization, Project administration, Supervision, Writing – original draft. HA: Writing – review & editing. LI-C: Writing – review & editing.
